# Does corporate governance and balance sheet feature influence the financial policy of cooperatives? PLS-SEM approach

**DOI:** 10.1371/journal.pone.0302121

**Published:** 2024-05-14

**Authors:** Ayalew Ali, Bayelign Abebe

**Affiliations:** 1 Department of Cooperative, College of Business & Economics, Mizan-Tepi University, Teppi, Ethiopia; 2 Department of Marketing Management, College of Business & Economics, Mizan-Tepi University, Teppi, Ethiopia; Yunnan Technology and Business University, CHINA

## Abstract

Effective financial policy minimizes business risk, increases the net present value of the Company’s investment programs and increases value for shareholders. However, the impact hasn’t yet been examined in the research area. The purpose of this study is to empirically investigate, how corporate governance and balance sheet aspects affect the financial policy of cooperatives in south-western Ethiopia using the PLS-SEM model. Information covering three years from 2020 to 2022 was gathered from 145 cooperatives. The study used corporate governance and balance sheet features as the latent factors that affect the dependent variable cooperative financial policy measured by both short-term debt and long-term debt. Managerial characteristics were used as the control variables. The study discovered that corporate governance has negative and significant effect on the financial policy of cooperatives in southwest Ethiopia. The study also revealed that balance sheet features have significant and positive effect the financial policy of cooperatives in southwest Ethiopia. Additionally, managerial characteristics’ have a significant impact on the financial policy and balance sheet features but have no impact on the corporate governance of cooperatives. The study concludes that the financial policy of cooperatives in southwest Ethiopia is significantly influenced by all aspects of corporate governance, balance sheet features, and management characteristics’. The study advises cooperatives to consider managerial characteristics’, corporate governance, and balance sheet characteristics while establishing their financial policy.

## 1. Introduction

Making a decision regarding a company’s financial policy is one of the most important aspects of managing a firm. A growing business normally needs funding, which can be provided by a combination of loan and stock [1]. Good financial decisions can increase shareholder value, reduce business risk, and increase the net present value of the company’s investment initiatives [2]. In addition to helping an organization maximize returns, a sound financial policy also strengthens its capacity to meet the demands of a competitive [3].

Finding the ideal financing mix that boosts shareholder value would be the responsibility of the finance management [4]. The changes in the range of market choices in the financial policy offer insightful information about the variables influencing those choices. The choice of financial policy is crucial for cooperatives because the operating cash flow is split between creditors and members, which can result in conflicts between members and managers. As a result, determining the optimal capital and making financial resource decisions are crucial issues that can directly affect cooperative economic activities and commercial connections.

The organizational structure of a company, which is characterized by the traditional conceptual separation of ownership and control, has a considerable impact on the financial policy of that company. There may be certain governance challenges, often referred to as agency problems, because of the roles/powers and conflicts of interest between the principle (owner) and the agent (manager). To prevent company collapse, financial crises, business failures, and bankruptcy fees, these disputes may be settled. The separation of ownership and control, along with the owners’ and managers’ divergent goals and informational inequalities, causes the company’s managers to act in their own self-interest [5–7].

Corporate governance refers to the systems, processes, procedures, responsibilities, and accountabilities [8]. The board of directors oversees management’s efforts to maximize shareholder value as part of a control and monitoring structure [9]. The ability of corporate governance to ensure legitimacy is one of the most significant aspects of ESG (environmental, social, and governance) indices [10–13]. Corporate governance attempts to make it easier to monitor and manage business operations effectively. Its core values include operating with fairness and transparency and improving disclosures to safeguard the interests of various stakeholders [14]. Furthermore, it is anticipated that corporate governance structures will improve the firm’s performance through sound decision-making [15].

The knowledge asymmetry between managers and shareholders is decreased by sound company governance. As corporate disclosure and transparency improve stock market liquidity, lowering transaction costs for the firm’s equities, it lowers the cost of capital for the company [16]. Due to agency problems that can be costly for the company by raising its cost of capital and ultimately requiring it to adjust or change its financing mix, corporate governance and financial policy are linked [17–20]. In light of the foregoing discussion, we contend that corporate governance influences financial policy directly and may also indirectly affect enterprises’ financing decisions by lowering the cost of capital.

Financing decisions are particularly pertinent and have a direct impact on the value of cooperatives and their members since they can have a significant impact on several aspects of the future position of cooperatives. Board characteristics, including board size, meeting frequency, and duality, among others, influences how corporations choose to finance themselves [21]. Additionally, the profitability, size, growth potential, and tangibility of assets on balance sheets and income statements, as well as the non-debt tax shield, volatility, and liquidity, have an impact on a company’s financial policy [22]. It is crucial to examine whether corporate governance and balance sheet features might support or undermine cooperatives’ financial policies.

Undercapitalization and financial policy are two issues that cooperatives deal with [23]. Due to low financial resources and relatively high capital costs, which deprive members’ profit, and investment? The capital limits cooperatives face and their detrimental consequences on the assets of members have been clarified by a number of arguments, according to [24]. One of the most significant of these is the structure of property rights and the incentives it provides to its members not to invest. Traditional cooperatives, in fact, are totally reliant on the money of their current members to transact, which restricts the cooperative’s ability to raise investment capital from any potential institutions.

According to earlier models [25] cooperative finance policies perform worse than those of investor-owned businesses. Less "tradability" of ownership rights or a fixed capital allocation to the cooperatives’ pool of capital is thought to be the main causes of this imbalance. This essay makes the case that there are additional factors that may help to explain why cooperatives’ financial policies are less effective than those of investor-owned companies. These additional factors include cooperative corporate governance’s limited application and understanding as well as issues with financial statement and audit report preparation.

Additionally, there are theoretical and empirical studies on the impact of balance sheet features and corporate governance on firms’ financial policy; nevertheless, the majority of these studies used data from advanced economies. Moreover, the majority of the cooperatives in the study area do not prepare financial statements that are in violation of Article 50 of Cooperative Proclamation No. 985/2016 and are not audited. The impact on the financial policy of those cooperatives in the study area, despite their preparation of financial statements and audit inspections, is not well understood and has not been well researched. As a result, the researchers are motivated because it is unknown how corporate governance and balance sheet features affect the financial policy of cooperatives in south-west Ethiopia. In this sense, it would be important to conduct empirical research to assess how company governance and balance sheet features affect cooperative financial policy.

[26] researched on the impact of corporate governance on financial policy which uses a panel data set and utilized OLS estimation with the right tests for the two most common panel data models, fixed and random effect models and found corporate governance mechanisms’ have significant effect on the financial policy. [27] Investigate the connection between corporate governance and financial policy, and found that firms that have effective corporate governance procedures benefit from easier access to capital and lower capital costs. Furthermore, research on the impact of firm-specific (balance sheet) features on financial policy was conducted by [28, 29] and it was discovered that these factors had a significant impact on financial policy. Neither of these researches used latent variables to capture corporate governance and balance sheet features in a structural equation model (SEM) or combined corporate governance and balance sheet features to evaluate the magnitude of their impact on the financial policy.

This study therefore, adopts the partial least squared structural equation model (PLS- SEM), a statistical technique from path analysis, to ascertain the impact of corporate governance and balance sheet aspects on cooperatives’ financial policy. The use of numerous predictors and standard variables, the creation of latent (unobservable) variables, the modeling of measurement errors for observed variables, and the testing of mediation and moderation interactions within a single model are all made possible by SEMs, according to [30], giving flexibility for testing such models. Therefore, the researchers’ conduct their research entitled as Does corporate governance and balance sheet feature influence the financial policy of cooperatives? PLS-SEM approach. Based on the above mentioned gaps on the effect of corporate governance, managerial characteristics, balance sheet features and financial policy the current study developed the following objectives

1. To investigate the effect of corporate governance on the financial policy of cooperatives in South west Ethiopia.
2. To assess the effect of balance sheet features on the financial policy of cooperatives in South west Ethiopia.

Consequently, the current paper seeks to make the following contributions to the existing literature: First, the study provides evidence on the relationship between corporate governance and balance sheet features on the financial policy of insurance companies in South West Ethiopia. Second, the findings are of interest to standards setters, regulators and shareholders to provide adequate standards that regulate the financial policy. Third, the study provides insight to managers of cooperatives and other stakeholders with suggestions on how to improve the financial policy of cooperatives.

The remainder of this paper is organized as follows. Section 2 presents the literature review and develops the hypotheses; Section 3 describes the research method; Section 4 reports result and discussions; the last section concludes the paper and offers suggestions and directions for future research.

## 2. Literature review

### 2.1 Theoretical literature review

#### 2.1.1 Pecking order theory

According to the pecking order theory, debt isn’t predefined; rather, it states that businesses have a distinct preference for using internally created assets over externally created ones, and businesses should follow a clearly indicated request of need as for a source of financing to reduce information asymmetry costs by first choosing held income, then obligation. The theory also assumes that as businesses grow financially stable and advantageous, they look less for externally created assets because they have enough internal assets to support their undertaking [31].

#### 2.1.2 Modigliani and miller theory

According to Modigliani and Miller’s argument, the firm’s valuation was independent of its profit strategy and debt, leading to a situation known as debt immateriality. The idea demonstrates that an association’s value is unaffected by its financial policies under a few essential assumptions. In Modigliani and Miller’s theory, the capital market is believed to be flawless because insiders and untouchables have unrestricted access to information, there is no need for exchange fees or tax collection, value and obligation decisions are no longer necessary, and both internal and external funds can be used in full substitution [32]. In addition, according to the Modigliani-Miller theorem (M&M), a company’s market value is accurately determined as the present value of its projected future earnings and its underlying assets, and is unrelated to its financial strategy.

#### 2.1.3 Trade off theory

According to the trade-off theory, organizations’ have the perfect debt and are moving in the right direction. It also emphasized the need for a compromise between the two since when the duty is used to incur debt, businesses must deal with the challenges of tax cuts and liquidation costs. According to the tradeoff theory, businesses with significant room for growth should purchase less because they stand to lose out financially. This is because the tradeoff hypothesis suggests that safe firms will exist. Therefore, companies with greater financial resources and more protected salaries should have higher obligation ratios. Companies having more intangible assets, whose value would be lost in the event of liquidation, must rely more on value finance. Tradeoff theory, in terms of gainfulness, more productive enterprises should imply a larger duty serving limit and more accessible wage to shield, which will result in a higher obligation proportion [33].

#### 2.1.4 Agency theory

According to agency theory, the best approach to debt management is to minimize office expenses by giving company executives more responsibilities or taking on more responsibility in order to kern directors’ propensity for excessive advantage use [6]. The important connections between principals and their relative agents are explained by the idea of agency theory. The principal, in the most basic sense, is someone who heavily relies on an agent to carry out specified financial decisions and transactions with potentially variable results.

#### 2.1.5 Resource dependency theory

According to the notion, a company’s board is crucial because it gives resources to managers, who then use them to accomplish organizational goals [34]. According to the theory, the board should support the executives’ financial, human, and intangible assets. To help executives enhance their abilities and performance, board members with experience and knowledge should mentor and train them. Board members can bring in crucial resources by connecting the organization with their own networks. The argument holds that the CEO should have the authority to make the majority of company decisions, with some going to the board for approval. According to [35], the goal of stakeholder theory in the banking sector is to satisfy depositors, owners, and other key stakeholders through an efficient governance system that fosters trust and transparency.

### 2.2 Empirical literature review and hypothesis development

#### 2.2.1 Corporate governance and financial policy

The choice of debt structure is crucial since it has a direct impact on a company’s profitability [36]. Moreover, [37] concluded that the capital structure has a significant effect on cooperatives financial performance. According to [38], the effective gathering and use of resources is one of the key components of the company’s financial plan. [39], state that the presence of a well-developed capital market, financial intermediary, corporate governance, and legal stability offered by a government all encourage debt efficacy. A corporate organization’s financial situation will depend on the resources it owns and the responsibilities it must fulfill [40]. Additionally, [27] contend that strong corporate governance arrangements assist businesses by facilitating quicker access to capital and lowering capital expenditures. The following corporate governance characteristics affect debt policy:

*2*.*2*.*1*.*1 Board size*. The size of the board has a significant impact on how companies handle debt. According to the notion of resource dependence theory, a larger board will benefit the organization by providing a network to the outside community and ensuring a larger resource base [41, 42]. Similar to this, [43] assert that larger boards aim for lower debt levels than small boards. The impact of corporate governance practices on insurance businesses in Nepal was examined by [44], which discovered that large boards can enhance board independence and diversity, which in turn improves firm performance. Additionally, a study by [45, 46] on the effect of corporate governance on the performance of insurance companies found that board size has a positive effect.

Additionally, [47] carried out a study to look into the impact of corporate governance on capital structure among Malaysian public listed businesses. This analysis discovered a strong correlation between board size and the debt to equity ratio. According to [3, 48, 49] found that, board size has significant and positive effect on debt ratio. They reasoned that large boards would lead to better decision-making due to knowledge, skill, and a diversity of viewpoints among the board of directors, which would lead to better investment decisions, and that large boards would make companies more efficient since they would use high debt to increase the firm’s value. The aforementioned justifications suggest that board size will affect how policies are created. Therefore, based on the resource dependency theory and empirical evidences this study developed the hypothesis as;

**H1**: Board size has positive and significant effect on the financial policy of cooperatives in south west Ethiopia.

*2*.*2*.*1*.*2 Board meeting*. In corporate governance, the term "meeting" relates to how frequently the boards convene each year. The agenda of board meetings should be designed to deal with routine financial and operational reports quickly while allowing enough time for discussion of strategic issues. Boards should routinely meet in executive session without management present to discuss issues that may be particularly delicate in terms of management. Additionally, more frequent board meetings will result in better management oversight, which will improve business performance [50]. Daily meetings, according to another argument, give directors more time to talk, create priorities, and assess management performance [51]. This will put managers in a better position to address emerging crucial issues in a timely way by enabling them to keep informed and aware of major developments inside the organization [1]. [52], who examined the significant mean difference between corporate governance practices in the financial policy and recommended applying corporate governance practices to the debt of the listed manufacturing companies, corroborated this finding. Based on the above empirical arguments this study developed the hypothesis as;

**H2**: The frequency of board meeting has significant and positive relationship with the financial policy of cooperatives in south west Ethiopia.

*2*.*2*.*1*.*3 Board gender diversity*. Due to higher quality earnings, increased accountability, and improved public information provision, a gender-diverse committee reduces information asymmetry. As women in the boardroom contribute to stronger scrutiny and consequently lower agency expenses, a gender balanced board has an influence on agency issues that is comparable to that of a high leverage. A gender-diverse board has a greater impact on low-debt companies than it does on high-debt companies. This claim is supported by the fact that alternatives such as debt financing and a board with a varied representation of genders are available. Results by [53] from the United Kingdom give evidence of differences between high-level debt and low-level debt enterprises with regard to the influence of a gender-diverse board on profits management, which lend support to this argument. According to their research, independent and female directors are beneficial to low-level loan enterprises. Additionally, [20] discovered that gender diversity on boards had a considerable impact on organizations’ financial policies. Thus, based on the agency theory and empirical evidences this study developed the hypothesis as;

**H3**: Board gender diversity has significant and positive effect on the financial policy of cooperatives in south west Ethiopia.

#### 2.2.2 Balance sheet features and financial policy

Assets, liabilities, and shareholder equity are all listed on a company’s balance sheet. Additionally, a balance sheet gives a snapshot of the financial situation of a corporation at any one time. [28, 29] claim that balance sheet features have an impact on a company’s debt policy [54]. Furthermore, balance sheet features have also significant impact on the dividend policy. The following explanatory variables are used in this paper as the aspects of the balance sheet that have an impact on cooperatives’ financing policy.

*2*.*2*.*2*.*1 Asset size*. The majority of empirical investigations, including those by [55, 56], found that there was a correlation between debt and asset size. [57] Found a similar correlation between asset size and rate of debt change. The similar favorable correlation between asset size and long and short- term debt was discovered by [58, 59]. Found a correlation between long-term debt and asset size that was positive. [60] came to the same conclusion about the long-term debt relationship, but they deduced that there was a negative correlation between short-term leverage and asset size. [61] Found a correlation between firm size and debt ratio in Malaysia. Additionally, [62] discovered that asset size has a significant impact on the monetary policy of commercial banks in the emerging market. Therefore, based on the above empirical arguments this study developed the hypothesis as;

**H4**: Asset size has significant and positive effect on the financial policy of cooperatives in south west Ethiopia.

*2*.*2*.*2*.*2*. *Liquidity*. The main element thought to have an impact on a company’s financial policy is liquidity. The majority of empirical evidence suggests a significant relationship between liquidity and financial policy [63]. Shown that relying on long-term debt to finance expansion is essential to maintaining higher liquidity levels. Again, according to trade-off theory, there is a positive correlation between debt ratio and liquidity since high liquidity firms can pay short-term creditors on time [64]. Found that financial policy and liquidity are positively correlated. This is because high-liquidity enterprises should choose debt as a significant component of their capital structure. Highly liquid corporations should use money created internally that do not include the fixed payment of interest [65–67]. A positive association between liquidity and financial policy was also discovered by [62]. Thus, based on tradeoff theory and empirical arguments this study developed the hypothesis as;

**H5**: Liquidity has a significant positive relationship with the financial policy of cooperatives in south west Ethiopia.

*2*.*2*.*2*.*3*. *Asset tangibility*. The change in asset structure/tangibility would change the long-term debt ratio according to the tradeoff theory, free cash flow theory, and pecking order theory. According to these theories, expanding organizations’ prefer to use debt to finance investors, whereas mature firms typically utilize internal funds. This is because mature firms tend to employ more tangible assets, while rising firms tend to use fewer tangible assets. The idea of trade-offs predicts a significant link between tangibility and debt ratio. According to [68], the reason for the positive association is that a company with a lot of fixed assets may readily collect the loan at cheaper rates due to the tangibility (collateral value) of the fixed assets. Asymmetric information exists in the rapidly evolving world of today, when companies with bigger fixed assets can readily borrow loans because it is highly accepted as a security for creditors. Therefore, based on tradeoff, free cash flow, and pecking order theories and empirical arguments this study developed the hypothesis as;

**H6**: Asset tangibility has significant and positive effect on the financial policy of cooperatives in south west Ethiopia.

### 2.3 Conceptual framework

**Fig 1:**
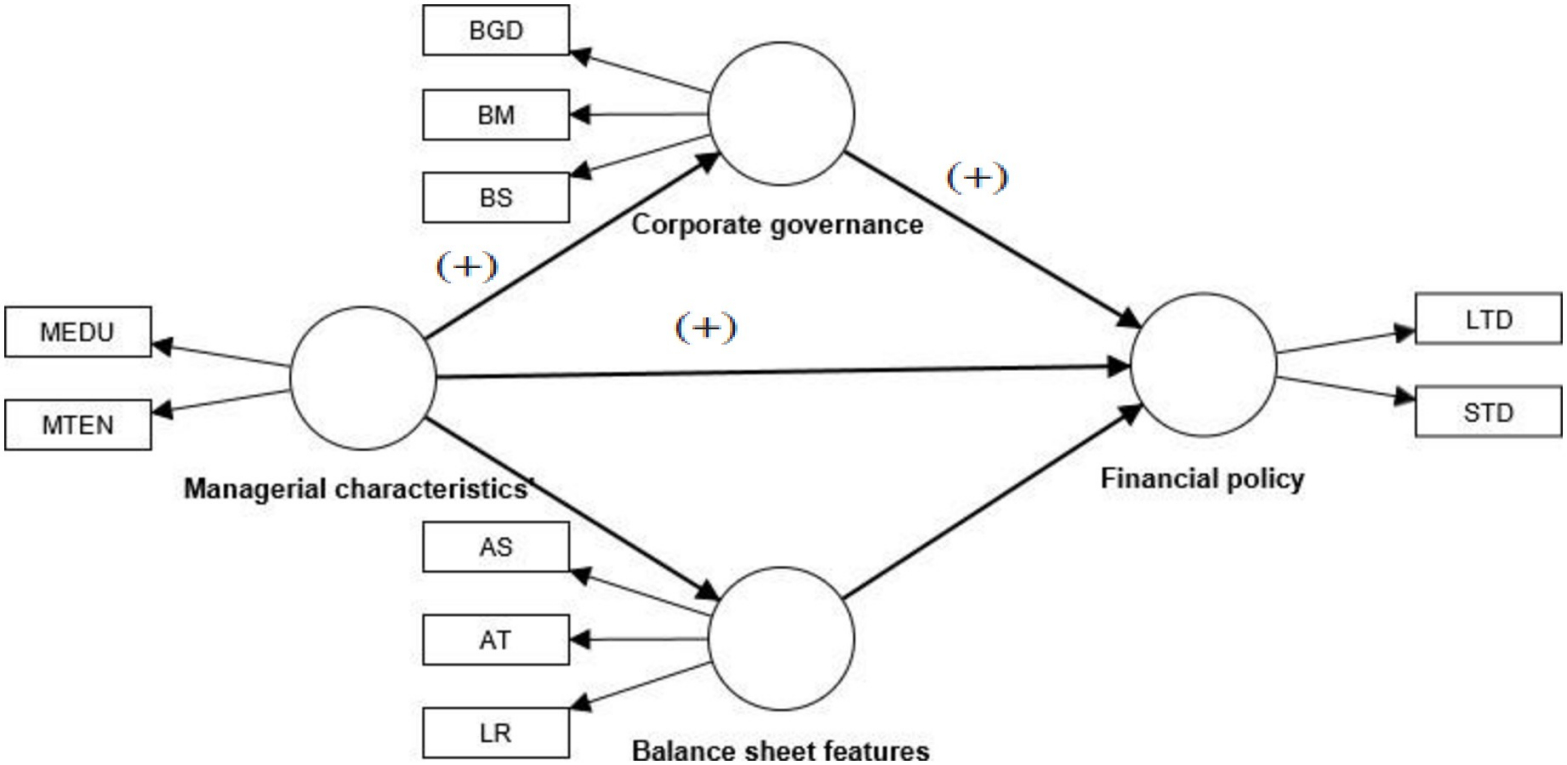
Conceptual framework. **Source**: SMART PLS-SEM.

Fig 1 below shows the relationship between the study’s dependent and independent variables. Financial policy is the dependent variable, and it is assessed by both long-term debt and short-term debt. As latent variables, asset size (AS), liquidity (LIQ), and asset tangibility (AT), bored size (BS), board meeting (BM), and board gender diversity (BGD) are used to predict corporate governance and balance sheet aspects, respectively. Moreover, managerial characteristics’ variables managers’ education and managers’ tenure are used as the control variables.

## 3. Method and material

### 3.1 Research design

The main goal of this study is to determine how corporate governance and balance sheet features affect cooperatives financial policy. Explanatory research method was used to determine and assess the cause-and-effect relationship between the variables being taken into consideration in order to accomplish this goal. A descriptive survey research design was also used because the questionnaires were delivered to the board of directors.

### 3.2 Research approach

There are three main types of research methodologies. These research methodologies include mixed, qualitative, and quantitative. A method for investigating and comprehending the meaning that individuals or groups assign to a social or human situation is qualitative research. A method for testing objective hypotheses by looking at the relationship between variables is quantitative research. To enable statistical analysis of numbered data, these variables can be measured, often using instruments. A technique to enquiry known as a "mixed research approach" entails gathering both quantitative and qualitative data, combining the two types of data, and employing various designs that may include philosophical presumptions and theoretical frameworks. This study used a mixed research method.

### 3.3 Source of data and collection methods

The research employed both primary and secondary data. The secondary sources of data are from sampled cooperatives’ audited financial accounts throughout the years (2020–2022). Only three years data of cooperatives is used because the cooperatives in the study area do not have organized consecutive year audited financial statement. Since boards of directors are in a better position to have all information pertaining to corporate governance of cooperatives, they provided the primary data through questionnaires.

### 3.4 Population of the study and sampling design

The major goal of the study is to determine how the financial policy of cooperatives in south- west Ethiopia is impacted by corporate governance and balance sheet features. Currently, there are 673 primary cooperatives serving cooperative members and society at large, according to the Bench Sheko, Sheka, and West Omo zones cooperative offices. Primary cooperatives in the Bench Sheko, Sheka, and West Omo zones were the study’s target populations. The principal cooperatives running in the Bench Sheko, Sheka, and West Omo zones made up the sample. From a total of 673 primary cooperatives, a pilot survey has been conducted from each worda, and the researchers have purposefully chosen 145 primary cooperatives that will be utilized as representatives. The availability of three consecutive years’ of financial reports is the standard that is utilized to purposefully choose primary cooperatives. This suggests that main cooperatives were chosen as a sample since they prepared and still have financial accounts from the year between 2020 and 2022.

### 3.5 Structural equation modeling

SEMs offer flexibility for testing such models by allowing the use of multiple predictors and criterion variables, the construction of latent (unobservable) variables, modeling measurement errors for observed variables, and testing mediation and moderation relationships in a single model, according to [30, 69–71]. SEM covers all reflected indicators under a single architecture. Partial least squares structural equation modeling (PLS-SEM) and covariance- based structural equation modeling (CB-SEM) are the two types of SEM used in research. Due to theoretical and methodological issues, PLS-SEM has gained greater traction than CB-SEM [72, 73]. The PLS-SEM methodology is very useful when working with really complex data. This methodology estimates latent variables by using composites, which are exact linear combinations of the indicators provided to the latent variables [30].

In light of this, the partial least-squares structural equation modeling (PLS-SEM) methodology was used to examine the impact of corporate governance and balance sheet features on the financial policy of cooperatives in south-western Ethiopia. The corporate governance, balance sheet features, and financial policy are frequently latent and cannot be detected right away, so the PLS-SEM approach was chosen because, financial policy cannot be directly quantified until more than one financial policy proxy is utilized. On the other hand, corporate governance and balance sheet features are measured using a variety of metrics. Latent variables include company governance, financial policy, and balance sheet features. Smart-PLS 4 software was used to implement PLS-SEM since this technique effectively handles nonlinear interactions. Since all the dependent and independent variables (corporate governance, financial policy and balance sheet features) are measured using more than two proxies PLS SEM is appropriate model for this study that is supported by [30].

### 3.6 Variables and measures

Following other research [74, 75] long-term debt (LTD) and short-term debt (STD) were employed as the dependent variables to measure the financial policy. Board meeting frequency, board gender diversity, and board size are used as independent variables. Similar to the previous study, the cooperative balance sheet features asset size, liquidity, and asset tangibility were used once more as the explanatory factors that can influence the cooperative financial policy. Additionally, the managers’ tenure and their degree of education act as moderating factors that influence cooperatives’ financial policies. Table 1 below discusses the study variable symbols and measurements.

**Table 1:** Dependent and independent variables.

Variables	Observed variable	Proxy
**Dependent variable**	Long term debt	Long term debt to total assets
	Liquidity	Current assets to current liabilities
	Short term debt	Short term debt to total asset
**Corporate governance**	Board size	Number of boards in cooperatives
	Board meeting frequency	Number of meetings per year held by the board of directors in cooperatives
	Board gender diversity	Dummy 1 if there is gender diversity 0 other wise
**Balance sheet features**	Asset size	Natural log of total assets of cooperatives
	Liquidity	Current assets to current liabilities
	Asset tangibility	Fixed assets to total assets
**Moderating variables**	Managers tenure	Number of years managers has in cooperatives
	Managers education Level	The education level of managers in cooperatives

*Source*: Own design 2023

## 4. Result and discussions

### 4.1 PLS- SEM results

The stochastic multiple regression imputation procedure is used as the initial step in PLS-SEM to impute missing data. The reflecting measuring scales that make up the latent constructs are interchangeable and require a high degree of correlation. The loadings of all the variable indicators in the constructs are utilized for scale purification in the model’s initial evaluation. Any indicator with a loading of less than 0.5 is removed from the model. As a result, the indicator cannot be dropped because it differs from the 0.5.

### 4.2 Internal consistency reliability assessment

The "Cronbach’s alpha" is typically used to gauge internal consistency reliability, but with PLS- SEM, it tends to give a conservative reading [76]. Claim that earlier literature has recommended using composite reliability as an alternative. In light of this context, Table 2 in the study presents the composite dependability. In exploratory research, the acceptable range for composite reliability values is 0.60 to 0.70, and in more advanced stages of study, 0.70 to 0.90. The latent variables are reliable, as evidenced by Table 2 composite reliability score, which ranges from 0.725 to 0.883 for the entire latent construct. The latent variables are kept in the model since the average variance extracted (AVE) value is greater than 0.5 and the construct qualified composite reliability test both pass. Once more, Table 2 displays the indicator reliability, which is essentially the loading square. It is clear that every indicator’s reliability value is significantly higher than the lowest permissible level of 0.4 and very nearly at the desired level of 0.7.

**Table 2:** Reliability and validity of latent construct.

Latent variables	Indicators	Loading	Indicator reliability	T-test	P- va	Composite reliability	Average variance extracted AVE
Financial policy	LTD	0.989	0.742	1.217	0.041	0.741	0.721
	STD	0.781	0.693	1.315	0.031	0.751	0.832
Corporate governance	BS	0.926	0.662	2.311	0.034	0.821	0.693
	BM	0.791	0.814	1.712	0.000	0.725	0.701
	BGD	0.791	0.723	2.343	0.012	0.824	0.762
Balance sheet features	AS	0.772	0.811	0.214	0.032	0.883	0.831
	LIQ	0.871	0.721	1.331	0.047	0.821	0.762
	AT	0.823	0.72	1.421	0.022	0.767	0.741
Managerial characteristics’	MEDU	0.793	0.744	2.462	0.013	0.821	0.732
	MTEN	0.948	0.823	1.215	0.031	0.772	0.781

***Source***: SMART PLS 4 result

### 4.3 Convergent validity

It is important to confirm the conceptual validity of each variable AVE, claims [77]. Convergent validity is proven if all AVEs are higher than the cutoff of 0.5. All of the AVEs in Table 2 above are more than 0.5, confirming the convergent validity.

### 4.4 Discriminant validity

According to [78], discriminant validity attests to a construct measure’s empirical distinctiveness and verifies that it captures relevant facts that other measures in a SEM do not. The Fornell- Larcker criterion, which states that the square root of AVE must be greater than the correlation between the construct and every other construct in the structural model, is illustrated in Table 3 below. The correlations between latent variables are shown in Table 3 along with the square root of the AVE for each latent variable. Each latent variable’s AVEs may be found to be larger than the correlation of the latent variables, showing the latent variables’ discriminant validity.

**Table 3:** Correlation among latent variables.

	Balance sheet features	Corporate governance	Financial policy	Managerial characteristics’
Balance sheet features	**0. 65**			
Corporate governance	-0.346	**0. 545**		
Financial policy	0.884	-0.511	**0.524**	
Managerial characteristics’	0.153	-0.349	0.176	**0.648**

Sources: SMART PLS 4 result

### 4.5 Correlation matrix

The purpose of the correlation matrix is to evaluate the degree of correlation between the variables and assess the possibility of multiple correlations among the regressors. Additionally, it determines if the dependent variables and independent factors have a positive or negative connection. This is crucial because it demonstrates whether there is a connection between the financial policies, balance sheet features, and corporate governance indices. The result in Table 4 shows that there is no multicollinearity and that the extents of correlation among the regressors were quite low. The outcome demonstrates that there is a considerable positive relationship between financial policy, managerial characteristics’, and all of the balance sheet features. The financial policy of cooperatives has, nevertheless, a negative correlation with characteristics related to corporate governance.

**Table 4:** Correlation matrix.

	LTD	STD	AS	LR	AT	BS	BM	BGD	MEDU	MTEN
LTD	1.000									
STD	0.03	1.000								
AS	0.231	-0.08	1.000							
LR	0.37	-0.02	0.250	1.000						
AT	0.05	0.070	-0.025	0.171	1.000					
BS	-0.20	0.030	-0.391	-0.208	-0.065	1.000				
BM	-0.11	-0.03	0.051	0.101	-0.017	0.020	1.000			
BGD	-0.04	0.052	0.053	0.012	0.010	-0.088	0.029	1.000		
MEDU	0.04	0.070	0.063	0.053	0.092	-0.117	-0.003	0.002	1.000	
MTEN	0.07	-0.02	-0.001	-0.064	-0.120	0.135	-0.102	0.022	0.106	0.001

***Source***: SMART PLS 4 result

### 4.6 Results of the measurement model of PLS-SEM

**Fig 2:**
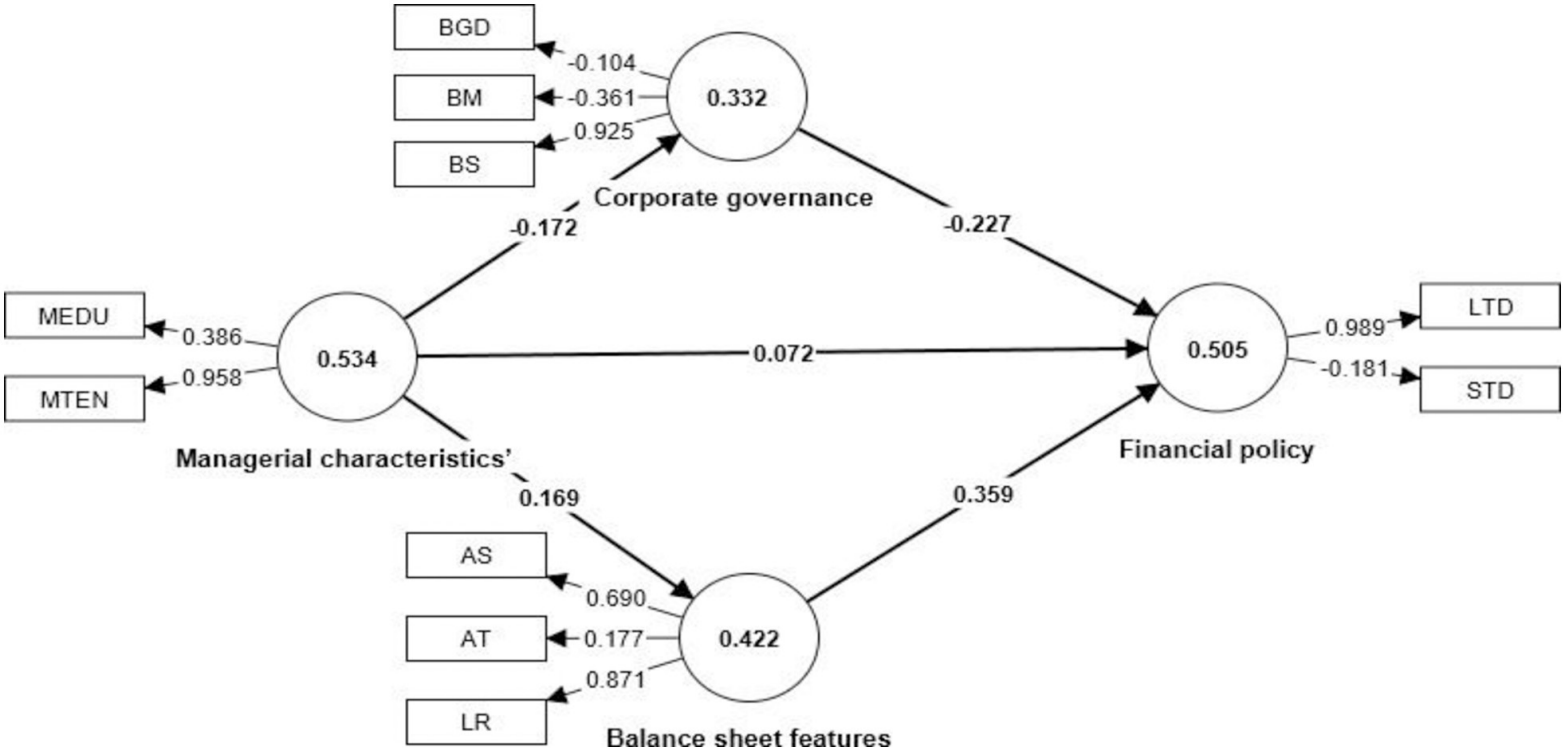
Result of linear boot strapping path coefficient. **Source**: SMART PLS-SEM result.

Numerous methods are used to estimate the measurement model’s path coefficient in order to guarantee the stability of the link between the latent variables. The use of exponential smoothing formulas is directly acknowledged by [79], which saying that this steady method yields estimates of the real standard errors that are consistent with those derived using bootstrapping. When the linear models are verified using PLS regression and robust path analysis, it has been demonstrated that this method produces more accurate estimates of the real standard errors. However, the significance and the R^2^ are shown in Fig 2. The model-wise dependent latent variable R^2^ demonstrated that 50.5 percent of the financial policy of the cooperatives in south- west Ethiopia is explained by both corporate governance and aspects of the balance sheet. On the other hand, the management characteristics’ factors account for 42.2% of balance sheet features and 33.3% of corporate governance. This indicates that corporate governance is a substantially poorer predictor of balance sheet features than the managerial characteristics’ variables considered.

### 4.7 Regression results and discussions

The discussion and analysis of the data in Fig 2 and Table 5 are presented in this section. According to Table 5 structural path significance in bootstrapping, the financial policy of cooperatives in south-west Ethiopia showed a statistically significant positive association with balance sheet features, with a coefficient of 0.359 and a p-value of 0.000. This is because cooperatives with large balance sheet features (asset size, liquidity, and asset tangibility) can practice economies of scale, have better business awareness, and can recruit better managers, and large sizes can allow greater specialization. Additionally, by locating external sources of finance for their operations, cooperatives with substantial balance sheet features, more specifically asset size, can also determine market power or the degree of concentration in the industry. The findings of this study are in agreement with Espinosa, [80] but are at odds with [81], who contend that large organizations’ degrees of asymmetry are larger as a result of their sophisticated knowledge structures.

**Table 5:** Structural path significance in bootstrapping.

	Original sample (O)	Sample mean (M)	Standard deviation (STDEV)	T statistics (O/STDEV)	P- v
Balance sheet features -> Financial policy	0.359	0.371	0.059	6.252	0.000***
Corporate governance -> Financial policy	-0.227	-0.230	0.100	2.254	0. 024**
Managerial characteristics’ -> Balance sheet features	0.169	0.156	0.053	2.876	0.004***
Managerial characteristics’ -> Corporate governance	-0.172	-0.174	0.034	5.094	0.1300
Managerial characteristics’ -> Financial policy	0.072	-0.015	0.050	0.301	0.041**

***Source***: SMART PLS result

The finding of a substantial positive association shows that cooperatives with higher balance sheet features, particularly liquidity, are able to fulfill their contractual obligations and don’t need to turn to debt financing. More crucially, more liquidity would guarantee cooperatives can pay their short-term debts. It follows that the effect of liquidity has a bigger effect on cooperative financial policy, and the outcome is consistent with the static trade off theory. Overall, the positive impact of liquidity on financial policy choices suggests that cooperatives in south-western Ethiopia view liquidity as a safety net that will ensure they can continue to exist and carry out their obligations in situations where it is challenging for them to raise money, when their earnings are low, or when their capital costs are extremely high. However, the results of this study are at odds with those of [20, 82].

A corporation with a lot of fixed assets can easily collect the debt at reduced rates because of the collateral value of such fixed assets (tangibility), which is a benefit of balance sheet features proxied by asset tangibility on cooperatives financial policy. The static trade off argument is supported by the observation of a large positive link between the tangibility of assets and financial policy. Furthermore, because lending to financial institutions is frequently contingent on collateral, the findings of this study are compatible with the empirical finding of [68].

A statistically significant negative link between financial policy of cooperatives and corporate governance, as evaluated by board size, board meeting frequency, and board gender diversity, was found in Table 5 and Fig 2, with a coefficient of 0.227 and a p-value of 0.024. This implies that a weakening of the financial policy of cooperatives by 22.7 percent results from an increase in one unit of corporate governance of cooperatives. The negative relationship between board size and financial policy is because a larger number of boards results in less effective management oversight because more directors make it more difficult to work together and make decisions, and a larger number of boards further reduces the corporate debt ratio or increases risky assets. The findings of this study, which demonstrated that an organization with a bigger number of boards reduces its debt ratio and adds hazardous assets to improve its profitability, and financial policy, are supported by [80, 81]. This is due to the potential influence of larger senior executive boards on the effectiveness of monitoring efforts. These duties, such as planning, communicating, and making decisions, may be burdensome for larger boards of executives, which would reduce the effectiveness of their role.

In a similar vein, the negative impact of corporate governance on cooperatives’ financial policies suggests that the cooperatives in south-west Ethiopia have poor corporate governance that negatively impacts their financial policy and drives them into bankruptcy. The study’s findings are in line with those of [17–20] that support the idea that poor corporate governance may increase information asymmetry by weakening corporate disclosure practices and monitoring mechanisms, which ultimately increases agency costs and financial policy of the firms.

Table 5 also showed that the variables measuring managers’ tenure and education, which were connected to managerial characteristics’, had a significant negative impact on cooperatives’ corporate governance and a significant impact on balance sheet features. These factors thereby limit the impact of balance sheet characteristics on the financial policy of cooperatives in south- western Ethiopia. This implies that cooperatives in south-west Ethiopia with high levels of managers’ education (MEDU) and tenure (MTEN) are more likely to increase their balance sheets’ features, whereas an increase in both managers’ education and tenure has no impact on the corporate governance of those cooperatives. There is a statistically significant positive link with a coefficient of 0.072 and p-value of 0.041 between the financial policy of cooperatives and the management characteristics’ proxies. This indicates that an increase in the managers’ tenure and education improves cooperatives’ financial policies by 7.2%. The significant positive relationship between managers’ tenure and educational attainment and the cooperative’s financial policy suggests that highly educated managers favor the adoption of cutting-edge practices that have a direct impact on financial policy. The findings of this study agree with those of [20]. Additionally, the correlation between managers’ education and tenure shows that managers with higher levels of education and tenure have a better understanding of the industry and market, which results in superior monitoring. The results are in line with the resource dependency theory and [82] observation that managers with a lengthy tenure and a high degree of education are more likely to employ sound financial policy.

## 5. Conclusion

The innovative SMART PLS approach is used in this research to present the impact of corporate governance and balance sheet features on the financial policy of cooperatives in south-west Ethiopia. The study adds to the body of research by concentrating on an emerging country from 2020 to 2022 in an effort to find empirical information that may be used to solve the financial policy conundrum in a novel way. The study built a PLS SEM to investigate the impact of corporate governance and balance sheet features on the financial policy of cooperatives using information from the annual report and financial statements of cooperatives. Additionally, questionnaires were employed in the study to gather information about cooperative corporate governance. The study discovered that the financial policy of cooperatives in south-west Ethiopia is significantly and negatively impacted by corporate governance. The detrimental impact of corporate governance on cooperative financial policy revealed that corporate governance, as measured by board size, diminishes the effectiveness of monitoring operations, increases risky assets, and decreases the cooperative’s debt ratio. Similar to this, corporate governance has a negative impact since it drives up the administrative costs of cooperatives in the research area as measured by the frequency of board meetings.

Additionally, the study discovered that the financial policy of cooperatives in south-west Ethiopia is significantly and favorably impacted by balance sheet features. The significant impact of balance sheet features, such as asset size, liquidity, and tangibility, shows that cooperatives can take advantage of economies of scale, able to fulfill their contractual obligations, and conveniently collect the debt at cheaper rates, respectively. The study’s findings also showed that managerial characteristics’, as measured by managers’ tenure and education, have a significant impact on the financial policy and balance sheet features but have a negative impact on cooperatives’ corporate governance in south-western Ethiopia. The study comes to the conclusion that factors related to corporate governance, balance sheet features, and managerial characteristics’ have a substantial impact on cooperatives’ financial policy in south-western Ethiopia.

## 6. Policy implications

Based on the finding of the study the following policy implications were given: Cooperatives should refrain from electing large board sizes because lower board sizes actively monitor cooperative boards, which will increase the value of the cooperative and promote efficient financial policy. Therefore, cooperatives with strong corporate governance are the focus of creditors. In order for cooperatives to develop successful financial policy, the board should meet at least once each month during the fiscal year. However, the study area’s cooperatives had more meetings, which raised administrative costs. As a result, cooperative board members should hold fewer board meetings.

Moreover, in order to take use of young people’s skills, the board should have more young, freshmen members. This does not imply that all cooperative boards have long tenure; rather, it suggests collaboration between long-tenured boards and young managers. Board members in the study region were all men, in violation of Article 34, Subsection 7 of Cooperative Proclamation No. 985/2016, which states that at least 30% of the board members of every cooperative society must be women. Accordingly, the study suggests that cooperatives and cooperative development offices should encourage women to hold leadership positions and raise knowledge of leadership abilities by providing short-term courses to female members. On the other hand, Article (34) sub-article (7) of the cooperative proclamation must be observed and controlled by the cooperatives in the field of study and the promotion office.

In addition large cooperatives identify external sources of funding for their activities to determine market power or the degree of industry concentration. Because large cooperatives can take advantage of economies of scale, the study suggested that cooperatives grow the amount of their assets. Likewise, the cooperative financial policy is significantly benefited by liquidity. Therefore, it makes sense that the effect of liquidity will have a stronger impact on cooperatives’ debt financing. In order to fulfill their contractual obligations and avoid resorting to debt financing, cooperatives must increase their liquidity during the study period. Higher liquidity would also guarantee that cooperatives will fulfill their short-term obligation. Asset tangibility and cooperative financial policy have a strong favorable link. Because of the tangibility (collateral value) of their fixed assets, cooperatives with a lot of them can readily collect the loan at reduced rates. Cooperatives must raise the proportion of fixed assets in their total assets since higher fixed asset levels make it easier to collect debt at cheaper rates due to the tangibility and collateral value of those fixed assets. Education of managers is significantly and favorably related to the cooperative financial policy. The study concludes that cooperative should provide managers with current information on industry trends to aid in the formulation of effective financial policies.

The findings of the study are subject to limitations. First, the financial policy indicators selected for the study were limited to only debt policy however; financial policy is also proxied by, dividend policy and investment policy. Therefore, should be more research done in this area, taking into account investment and dividend policies. Second, the corporate governance related explanatory variables that have been used for this study where: board size, board meeting and board gender diversity and the explanatory variables used for balance sheet features are asset size, liquidity and asset tangibility. Those variables are not the only variables that affect the financial policy of cooperatives under the study area. Third, probability-sampling method was not applied to select the sample from the population, which reduces the sample bias. Finally, the results of this study are not generalized to other sectors other than cooperatives. Therefore, investigations will be made including other institutions.

## Supporting information

S1 Data (CSV)
